# Pre-Use Susceptibility to Ceftaroline in Clinical *Staphylococcus aureus* Isolates from Germany: Is There a Non-Susceptible Pool to be Selected?

**DOI:** 10.1371/journal.pone.0125864

**Published:** 2015-05-08

**Authors:** Birgit Strommenger, Franziska Layer, Ingo Klare, Guido Werner

**Affiliations:** National Reference Centre for Staphylococci and Enterococci, Robert Koch Institute, Wernigerode Branch, Wernigerode, Germany; Universitätsklinikum Hamburg-Eppendorf, GERMANY

## Abstract

Ceftaroline is a new cephalosporin active against Methicillin-resistant *Staphylococcus aureus* (MRSA). Based on a representative collection of clinical *S*. *aureus* isolates from Germany, supplemented with isolates of clonal lineages ST228 and ST239, we demonstrate the in-vitro susceptibility towards ceftaroline prior to its introduction into clinical use for a total of 219 isolates. Susceptibility testing was performed by broth microdilution, disc diffusion and Etest, respectively. Results were interpreted according to EUCAST guidelines and showed considerable variance in dependence on clonal affiliation of the isolates tested. Among isolates of widespread hospital-associated lineages we found a high proportion of clinical isolates with MICs close to the EUCAST breakpoint (MIC_50/90_ 1.0/1.5 mg/L); currently, interpretation of these “borderline” MICs is complicated by a lack of concordant susceptibility testing methods and reasonable breakpoint determination. Isolates of clonal lineages ST228 and ST239 demonstrated increased MIC_50/90_ values of 2.5/3.33 mg/L. Sequencing of *mecA* revealed no association of resistance to a specific mecA polymorphism, but rather reveals two regions in the non-penicillin-binding domain of PbP2a which displayed different combinations of mutations putatively involved in resistance development. This study provides national baseline data to (i) adjust susceptibility testing methods and current breakpoints to clinical and epidemiological requirements, (ii) evaluate current breakpoints with respect to therapeutic outcome and (iii) monitor further resistance evolution.

## Introduction

The worldwide emergence and spread of methicillin resistant *Staphylococcus aureus* (MRSA) over the last 50 years represents one of the most serious challenges to clinical microbiologists worldwide. Moreover, the evolution of several MRSA lineages towards resistance to additional antibiotic classes is a matter of growing concern [1]. This situation has been complicated even further by the recent occurrence and spread of community- and livestock-associated MRSA, nowadays invading also hospitals [2]. On the other hand we are observing stagnation in the development of new antibiotic agents for several years [3]. As a consequence clinicians and public health authorities might face an increasing number of serious staphylococcal infections which can only be treated with a limited number of antibiotics of last resort.

Ceftaroline, the active metabolite of ceftaroline fosamil, is a new bactericidal cephalosporin [4]. Ceftaroline and ceftobiprole, which are the only “fifth-generation” cephalosporins to date, both possess expanded gram-positive-including MRSA- activity [5]. MRSA, in general, are resistant to all previously available beta-lactams. Beta-lactam resistance is mediated by the expression of the *mecA*-encoded low-affinity penicillin-binding-protein 2a (PBP2a), which does not bind clinically used beta-lactams at therapeutically relevant concentrations. In contrast, ceftaroline binds to PBP2a with a significantly higher affinity. As a result of irreversible inhibition of cell wall synthesis ceftaroline exhibits an improved antimicrobial activity against MRSA both in vitro and in vivo [6]. Its binding affinity to the recently described, *mecC*-encoded, alternative PBP (LGA251) has not been assessed so far. Ceftaroline has been approved for the treatment of bacterial complicated skin and soft tissue infections (cSSTI) and community acquired pneumonia (CAP) in the U.S. and in Europe in 2010 and 2012, respectively. In Germany it became available for clinical treatment in October 2012. Susceptibility ranges and breakpoints were provided by CLSI [7] and EUCAST [8,9], but differences in breakpoint determinations complicate the evaluation of susceptibility studies currently conducted.

In several recent susceptibility studies from the U.S. and Europe ceftaroline demonstrates potent in-vitro activity against *S*. *aureus* clinical isolates, including MRSA, although the MRSA population, in general, showed significantly elevated MIC_50/90_ values [10,11]. Additionally, geographical differences regarding slightly elevated MIC_50/90_ values for MRSA in European and Asian/Pacific studies [11,12] to alarming ceftaroline resistance rates in studies from China were reported [13]. Against this background the major aim of this study was to determine the susceptibility towards ceftaroline in the clinical *S*. *aureus* and MRSA population in Germany prior to the introduction of this new compound into clinical use. The resulting data provide a basis for the evaluation of existing antibiotic susceptibility testing and interpretation guidelines, ongoing susceptibility surveillance projects and resulting treatment recommendations.

## Materials and Methods

### Bacterial isolates

*S*. *aureus* isolates originated from microbiological laboratories all over Germany and comprised phenotypically methicillin-susceptible (MSSA, MIC_OXA_ ≤ 2 mg/L, n = 27) as well as methicillin-resistant *S*. *aureus* (MRSA, MIC_OXA_ > 2 mg/L, n = 133). Non-duplicate isolates were collected at the German Reference Centre for Staphylococci and Enterococci from July to September 2012. They were selected to represent the current distribution of clonal lineages most prevalent in Germany at that time point [14,15]. The collection also included ten isolates possessing the newly described alternative *mec* gene, *mecC* [16], isolated from clinical infections in humans.

Since all isolates from clonal lineages ST228 and ST239 investigated initially revealed increased ceftaroline (CPT) MICs and previous studies already associated ST228 and ST239 isolates with increased ceftaroline MICs [17,18] we added all available isolates of clonal lineages ST228 (n = 34) and ST239 (n = 25) collected from January 2010 to September 2012 to the strain collection.

Isolates originated from skin and soft tissue infections (n = 78), bacteremia (n = 31), pulmonary tract infections (n = 15), urinary tract infections (n = 7), other infections (n = 4) and from samples of unknown origin (n = 28); the remaining 56 isolates were obtained from screening swabs; all isolates were cultured on sheep blood agar and confirmed as *S*. *aureus* by colony morphology and positive plasma coagulase reaction. Detailed strain data and results for all isolates investigated are summarized in S1 Table.

### Susceptibility testing

All isolates were subjected to susceptibility testing by means of broth microdilution (BMD) according to EUCAST (http://www.eucast.org/antimicrobial_susceptibility_testing/mic_determination). The following clinically or epidemiologically relevant antibiotics were tested and all results are summarized in S1 Table: penicillin (PEN), oxacillin (OXA), gentamicin (GEN), linezolid (LZD), erythromycin (ERY), clindamycin (CLI), tetracycline (TET), vancomycin (VAN), teicoplanin (TPL), ciprofloxacin (CIP), moxifloxacin (MFL), daptomycin (DAP), mupirocin (MUP), fosfomycin (PHO), rifampicin (RAM), fusidic acid (FUS), trimethoprim/sulfamethoxazole (SXT). CPT susceptibility was assessed by disc diffusion (DD, 5 μg-ceftaroline discs, MAST, Reinfeld, Germany), BMD and Etest. Ceftaroline was obtained by AstraZeneca (Wedel, Germany). All procedures were conducted according to EUCAST guidelines 2013 and including *S*. *aureus* ATCC 29213 as quality control strain [8]. Etest (Biomerieux, Nürtingen, Germany) was performed according to the manufacturer's instructions and using the same quality control strain. Ceftaroline susceptibility testing by DD and BMD methodology was done repeatedly (2 to 4 independent measures per isolate), and zone diameters and MICs were averaged for further analyses.

### Molecular strain characterisation

Genomic DNA was isolated from overnight cultures with the DNeasy Tissue Kit (Qiagen, Hilden, Germany) using lysostaphin (100 mg/L, Sigma, Taufkirchen, Germany) to achieve bacterial cell lysis. *spa*-typing was performed as described previously and isolates were assigned to clonal lineages using BURP [19]; for previously unknown *spa*-types multilocus sequence typing was performed for lineage allocation [20]. *mec* gene detection and sequencing of the *mecA* gene was performed using primers, PCR conditions and controls summarized in S2 Table. Results of molecular strain characterization, also demonstrating the “clonal representativeness” of the collection for the German *S*. *aureus* population, are summarized in S1 Table.

## Results

### Ceftaroline susceptibility of 160 *S*. *aureus* isolates representing prevalent clonal lineages in Germany

#### (a) MSSA

Among 27 MSSA isolates (MIC_OXA_ ≤ 2 mg/L) we found none exhibiting a mean inhibitory zone below 20 mm (EUCAST breakpoint, R < 20 mm); a single isolate exhibited a mean inhibitory zone of 20 mm and therefore had to be re-tested by a MIC-method according to the 2013 EUCAST guidelines. However, using BMD, all MSSA isolates had CPT MICs below or equal to 1 mg/L in at least 2 independent measurements. This also included 6 phenotypic MSSA which were found to be positive either for *mecA* (n = 4) or *mecC* (n = 2) but neither showed reduced inhibitory zones nor elevated CPT MICs. Etest results were in agreement with results of BMD, although Etest MICs were generally lower (one dilution step) in comparison to results obtained by BMD (Table 1).

**Table 1:** CPT susceptibility testing in different study populations using different methodologies and 2013 vs. 2014 EUCAST interpretation.

	MSSA (n = 27)		MRSA (n = 133)		ST228 (n = 34)		ST239 (n = 25)	
**zone diameter range (mm)**	20–35		16–30		16–30		17–35	
**S/R/retest according to EUCAST 2013** ^a^	27/0/1		122/11/51		7/27/8		9/16/12	
**S/R according to EUCAST 2014** ^b^	27/0		122/11		7/27		9/16	
	**BMD**	**Etest**	**BMD**	**Etest**	**BMD**	**Etest**	**BMD**	**Etest**
**MIC**_**50**_ **(mg/L)**	0.50	0.25	1.00	0.50	**2.00**	1.00	**2.50**	0.75
**MIC**_**90**_ **(mg/L)**	0.75	0.38	**1.50**	0.75	**3.33**	1.00	**3.00**	1.00
**MIC range (mg/L)** ^c^	0.16–1.00	0.09–0.75	0.31–4.00	0.25–1.50	0.38–4.00	0.19–1.50	0.33–3.33	0.19–1.00
**S/R according to EUCAST 2013/2014 ^a^^,^^b^**	27/0	27/0	85/48	128/5	3/31	33/1	4/21	25/0

^a^breakpoints EUCAST 2013: disc diffusion: inhibitory zone (mm) S≥20; R<20; to be retested by MIC method: 19–21 broth microdilution, BMD (mg/L) S≤1; R>1

^b^breakpoints EUCAST 2014: disc diffusion: inhibitory zone (mm) S≥20; R<20 broth microdilution, BMD (mg/L) S≤1; R>1

breakpoints CLSI: disc diffusion: inhibitory zone (mm) S≥24; I: 21–23; R≤20 broth microdilution, BMD (mg/L) S≤1; I = 2; R≥4

^c^MIC range tested: 0.016–16 mg/L CPT

#### (b) MRSA

Among 133 phenotypic MRSA (MIC_OXA_ > 2 mg/L) 131 possessed either *mecA* or *mecC* (see S1 Table). Eleven of these isolates showed mean zone diameters below 20 mm using DD. Fifty one isolates showed mean zone diameters between 19 and 21 mm (EUCAST 2013 breakpoint, R < 20 mm; 19–21 mm: to be retested by a MIC method). Among the 11 “DD resistant” isolates 4 strains had zone diameters smaller than 19 mm and three of these isolates revealed BMD MICs above 1 mg/L in several independent measurements (mean MIC 2 mg/L, n = 2; 4 mg/L, n = 1) indicating resistance according to EUCAST breakpoints; the fourth isolate revealed alternating MICs between 1 and 2 mg/L resulting in a mean MIC of 1.5 mg/L. Among the 51 isolates, which had to be retested by a MIC method according to 2013 EUCAST guidelines two isolates were repeatedly resistant (mean MIC 2 mg/L); 20 isolates exhibited a CPT sensitive phenotype with MICs below or equal to 1 mg/L, repeatedly (mean MIC ≤1 mg/L), and 29 isolates showed alternating MICs, resulting in mean MIC values between 1 and 2 mg/L.

**Fig 1:**
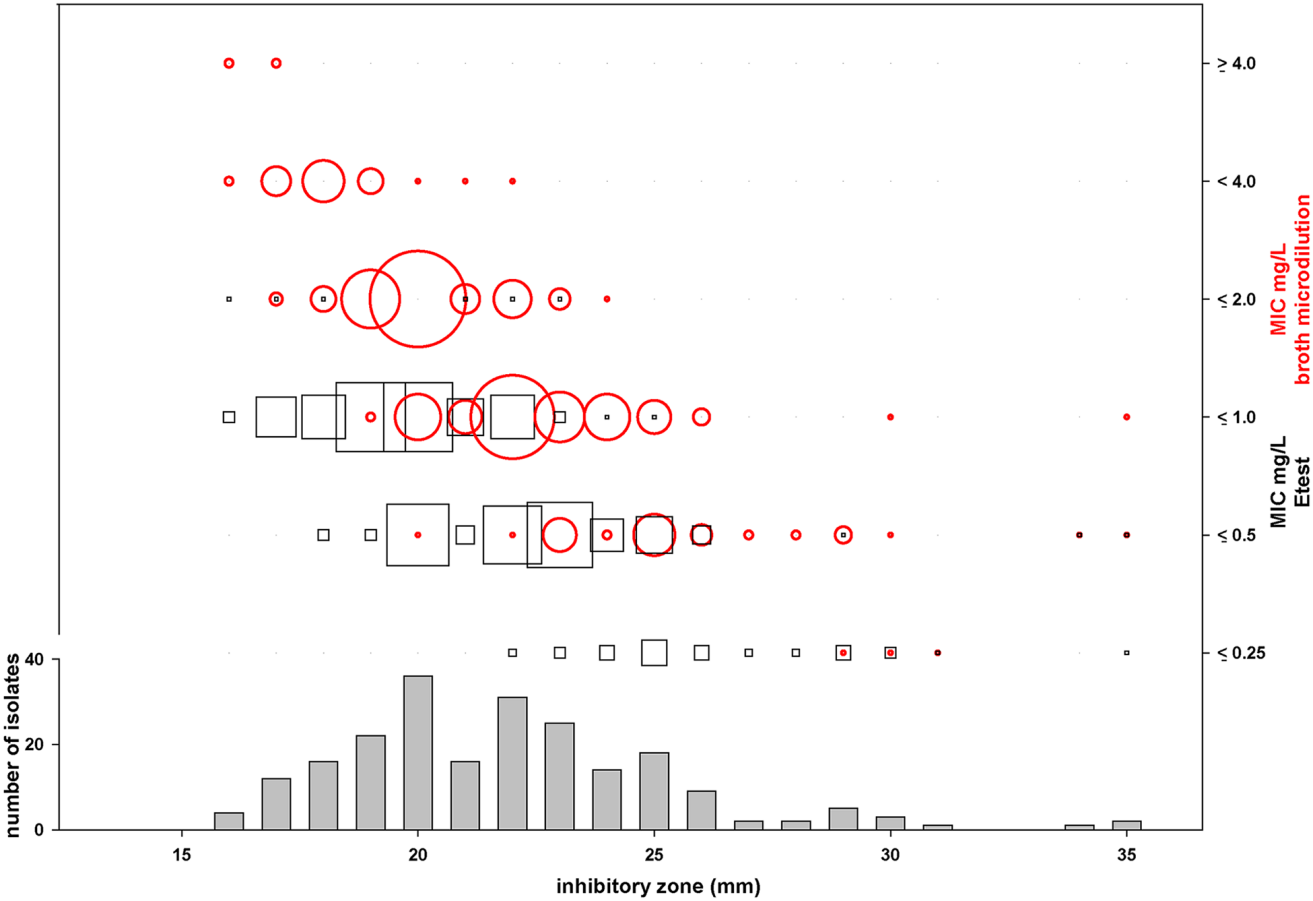
Comparative results of ceftaroline susceptibility testing for 219 *S*. *aureus* isolates described in this study using different test methodologies. Grey bars: agar diffusion, CPT discs (5 μg); black squares: CPT MICs determined by Etest; red circles: CPT MICs determined by BMD; The sizes of squares and circles denotes the number of isolates with the respective inhibitory zone/MIC combination.

122 isolates were “DD sensitive” with zone diameters larger than 20 mm and 78 of these revealed zone diameters above 21 mm; these 78 isolates included one isolate with a mean CPT BMD MIC of 2.5 mg/L (resistant according to EUCAST) as well as 65 repeatedly sensitive isolates (mean MIC ≤1 mg/L). Twelve isolates showed alternating MICs, resulting in mean MIC values between 1 and 2 mg/L (see S1 Table). CPT susceptibility data for MRSA are summarized in Table 1. BMD MICs correlated well with DD results with few exceptions as indicated in Fig 1. Moreover, BMD results appeared adequately reproducible with maximum one dilution difference between several independent measurements for individual isolates. Only for three isolates repeated BMD MICs differed by a range of two dilutions (see S1 Table). MICs were further determined using Etest, and in accordance to the results for MSSA, Etest MICs were approximately one dilution lower in comparison to results obtained by BMD (Table 1). As a consequence, only 2 out of 6 resistant isolates with mean MICs ≥ 2 mg/L (5 out of all isolates with mean MICs > 1 mg/L; n = 48) were detected by Etest (Etest MIC 1.25 and 1.5 mg/L, respectively; S1 Table).

### Ceftaroline susceptibility in isolates of clonal lineages ST228 and ST239

We investigated a total of 59 additional *S*. *aureus* isolates from clonal lineages ST228 and ST239, including four *mecA*-negative MSSA (OXA MIC ≤ 2mg/L); the 4 latter isolates were CPT sensitive with respect to all susceptibility test methods applied (DD: inhibitory zones 25–34 mm; BMD: mean CPT MICs 0.33–0.83 mg/L; Etest: mean CPT MICs 0.19–0.38 mg/L, S1 Table). In contrast, the majority of *mecA*-positive ST228 and ST239 isolates showed CPT non-susceptibility as demonstrated by DD and BMD results (Table 1). Seventy-three percent of these isolates (n = 43) showed zone diameters below 20 mm. Accordingly, 81% of the isolates (n = 48) displayed mean CPT MICs ≥ 2 mg/L (88%, 52 isolates with CPT MICs >1 mg/L), indicating individual MIC results between 2 and 4 mg/L in repeated measurements. Only 12% of isolates (n = 7, 4 *mecA* negatives) tested sensitive repeatedly (mean MICs ≤ 1 mg/L). The significant elevation of CPT MICs for ST228 and ST239 is also reflected by their MIC_50_ and MIC_90_ values (Table 1), however when using Etest, only one isolate was detected resistant (MIC 1.5 mg/L, Table 1).

### Molecular characterisation of *mecA* in ceftaroline-resistant and-sensitive isolates

We sequenced the *mecA* gene for a total of 58 geographically diverse isolates of clonal lineages ST228 and ST239, exhibiting different CPT MICs. For ST228 we found only one isolate that repeatedly tested sensitive with a mean MIC of 0.38 mg/L and three isolates which showed slightly elevated MICs resulting in mean CPT MICs below 2 mg/L; among ST239 isolates we found no repeatedly sensitive isolate, but three isolates with mean BMD MICs below 2 mg/L (S3 Table). Overall, we found 9 polymorphic loci within the *mecA* gene, including the amino acid positions M_122_, D_139_, N_146_, E_150_, N_204_, T_235_, E_239_, G_246_ and K_281_ all affecting the non-penicillin binding domain. The most common mutation was N_146_K which was found in 21 isolates from both clonal lineages; two additional substitutions were carried by isolates from both lineages, namely, E_239_K (4 isolates) and G_246_E (5 isolates, S3 Table). The substitutions M_122_I (n = 1, ST228), D_139_N (n = 16, ST228), E_150_K (n = 1, ST239), N_204_K (n = 16, ST239), T_235_I (n = 9, ST239) and K_281_R (n = 5, ST239) were found in one clonal lineage only.

## Discussion

The initial aim of this study was to evaluate the in-vitro susceptibility towards ceftaroline in the German clinial *S*. *aureus* population prior to introduction of this new cephalosporine into clinical use. It is well known that the *S*. *aureus* population structure varies with geographic location, with specific clonal lineages predominating in different areas of the world. Isolates of predominating lineages themselves are subject to continuous evolutionary processes, which lead to an accumulation of resistance traits most probably due to adaptation to local or regional antibiotic use [1]. On the other hand it was shown that the distribution of clonal lineages is constantly changing with newly emerging lineages replacing previously prevalent ones [15,21,22]. Driving forces for the appearance of such clonal waves are largely unknown, however, both processes lead to continuous changes in resistance and virulence properties of MRSA strains prevalent in hospitals and in the community which must be monitored to allow efficient therapy, infection control and prevention measures. Currently, the most prevalent hospital-associated clonal lineages in Germany are MRSA lineages ST225 and CC22. In addition, we are observing a multiplicity of various clonal lineages inside and especially outside the hospital [14,15,19]. Thus, in the first part of the study we aimed to establish a collection of isolates representing the current composition of the German clinical *S*. *aureus* population with respect to geographic origin as well as clonal distribution (S1 Table).

### Ceftaroline susceptibility in the German clinical *S*. *aureus* population before introduction of ceftaroline into clinical use

Among the 160 *S*. *aureus* isolates selected, as expected, all phenotypic MSSA were completely susceptible to ceftaroline as reflected by a MIC_50/90_ of 0.5/0.75 mg/L in repeated BMD experiments (Table 1). Among the phenotypic MRSA investigated we found a total of 63.9% of MRSA (85/133) to be susceptible repeatedly, whereas 4.5% of isolates (6/133) were resistant to ceftaroline in repeated experiments, with MICs of 2 to 4 mg/L. This resistance rate is similar to rates previously reported for isolates from cSSTIs from Europe [11] and to the published EUCAST data (http://mic.eucast.org/Eucast2/). The remaining 31.6% of isolates (42/133) showed altering MIC results ranging from 1 mg/L (susceptible according to EUCAST) to 2 mg/L (resistant according to EUCAST) challenging the fact that EUCAST—in contrast to CLSI—does not define an intermediate susceptibility range. Additionally, the current EUCAST breakpoint cuts through the MRSA “wildtype” MIC distribution [23] which might cause significant problems in laboratory susceptibility testing of ceftaroline. In the present study BMD MICs differed for almost all isolates by only one dilution which reflects the technical robustness of these results. However, the classification of these isolates as either CPT-resistant or-susceptible is debatable. Since our study only looked at microbiological strain properties we cannot comment on any putative therapeutic consequence; future clinical studies are required to elucidate clinical success rates for MRSA isolates with “borderline” CPT MICs. Therefore, it will be indispensable to include isolates representative for the clonal composition of the *S*. *aureus*/MRSA population in different geographical regions to suggest a “general” and more comprehensive clinical breakpoint.

The “MIC-shift” we observed for MRSA vs. MSSA is also reflected by increased MIC_50/90_ value for MRSA (Table 1) and is in accordance to previously published susceptibility data for the *S*. *aureus* populations, both in Europe and in the U.S. [11,24].

We found no significant difference in CPT susceptibility between *mecA-* and *mecC-*positive MRSA, suggesting that binding affinity of ceftaroline towards the distinct penicillin-binding proteins is comparable. This is in contrast to cefoxitin, where the reduced affinity of *mecC*-encoded PBP2a (LGA) results in increased cefoxitin MICs [25].

### Diagnostic challenges

At the present time none of the available semi-automated antibiotic susceptibility test systems includes ceftaroline for susceptibility testing. Thus, diagnostic laboratories might use DD methodology for ceftaroline susceptibility testing, initially. In this study we demonstrate that more than 30% of all clinical isolates show inhibitory zones of 19 to 21 mm. The majority of them belong to the MRSA lineages ST225 and CC22, which are highly prevalent in Germany (S1 Table). According to 2013 EUCAST guidelines [8], which were applied in this study, these isolates had to be “retested” by a MIC method. As a consequence, the clinical laboratory would have had to “retest” approximately one third of all isolates tested, which implies an enormous additional workload for daily routine. EUCAST addressed this problem in that retesting is no longer suggested in the current guidlines of 2014[9]. According to our data this strategy results in an underestimation of “borderline” resistant isolates, which display alternating CPT MICs between 1 and 2 mg/L. In the present study we found 52 isolates with zone diameters of 20 and 21 mm, respectively (Table 1); according to the current EUCAST guidelines these isolates are classified susceptible; however, regarding their BMD MICs, 7 of these isolates displayed CPT MICs of ≥ 2 mg/L and another 25 isolates showed alternating MICs between 1 and 2 mg/L. Currently, our knowledge about the clinical significance of these “borderline” resistant isolates is scarce; therefore MIC surveillance of respective isolates is crucial to monitor treatment efficiency and future development of CPT MICs.

Since individually manufactured BMD is usually not applied in routine microbiological laboratories, gradient strip based systems will most probably be used for MIC determination. However, our results strongly suggest, that gradient strip methods might generate MICs which are not concordant to those obtained by BMD. In this study Etest results revealed MICs which were on average approximately one dilution lower than those obtained by BMD (Table 1), thereby confirming previous reports [26]. This might lead to an underestimation of CPT resistance rates, especially in a geographical region with increased numbers of low-level resistant isolates (MICs 2–4 mg/L). However, the data presented here are limited as we did not use a variety of gradient strips from different suppliers which might result in divergent outcomes.

### Clonal lineages with reduced susceptibility

In our initial collection we found 6 isolates which were resistant towards ceftaroline, repeatedly. These isolates included all three isolates of clonal lineage ST239 as well as the single ST228 isolate present in our representative sample of 160 clinical isolates. Since previous studies associated ST228 and ST239 isolates with increased ceftaroline MICs [17,18] we supplemented our strain collection with isolates from both these lineages and found a significant elevation of CPT MICs for the majority of isolates (Table 1). Since isolates were collected between 2010 and 2012 we could infer that resistance had not been selected by the use of ceftaroline itself, but was present in these lineages before. It will be interesting to elucidate at what evolutionary stage non-susceptibility emerged and whether its emergence can be correlated with any selective pressure affecting these clonal lineages.

Neither ST228 nor ST239 are lineages highly prevalent in Germany; however, ST228 is widely disseminated in Southern and South East Europe [27–29] and occurs frequently also in neighbouring countries like Austria [30] and Switzerland [31]. Sporadic outbreaks were also reported from Germany [32]. ST239 is a globally occurring clone which is highly prevalent in countries in South East Europe and Asia [33–35]. In these geographic regions an increased ceftaroline resistance rate could be expected, and was documented recently for *S*. *aureus* from acute skin infections from China [13]. One could speculate that wider use of ceftaroline in future might select for these CPT resistant clonal lineages, thus contributing to future strain dynamics also in countries were ST228 and ST239 are not that prevalent today. Since these clonal lineages harbour more co-resistances than current successful epidemic strains, their spread would restrict treatment options for associated MRSA infections considerably.

### Molecular correlates of ceftaroline resistance

In a previous study Mendes et al. associated increased ceftaroline MICs with alterations in PBP2a, which appear to have contributed to the reduction in binding affinity towards ceftaroline [17]. They found mutations in both, the non-penicillin-binding and the transpeptidase domain of the protein thus suggesting that the accumulation of mutations in various parts of the PBP2a might contribute to a stepwise increase in MICs similar to mechanisms demonstrated for resistance towards ceftobiprole in *mecA*-containing *S*. *aureus* [36]. This hypothesis was recently corroborated in a structural study by Alm et al. [37]. However, all studies included only a very limited number of isolates. In this study we were able to investigate the *mecA* gene of 58 isolates assigned to clonal lineages ST228 and ST239, and showing different CPT MICs. Mutations associated with a CPT MIC increase should be present in all resistant isolates independent of clonal lineage or SCC*mec* type and absent in isolates which were repeatedly sensitive towards ceftaroline. Based on this hypothesis two amino acid alterations, N_146_E and E_239_K, draw our first attention. However, N_146_E was absent in 6 out of 8 ST239 isolates with elevated MICs. The alteration E_239_K was excluded as a sole reason for resistance since it occurred in only 4 isolates (mean MICs 2.0 to 3.0 mg/L). The third alteration present in both ST228 and ST239, G_246_E, was dismissed since it was present in two completely sensitive isolates (reference strain COL and 10–03087). The same was previously reported for the alteration N_204_K which also occurred in sensitive isolates [17]. As a consequence we conclude that low level ceftaroline resistance may be mediated by various mutations in two “hot spot” regions of the PBP2a, the non-penicillin-binding domain comprising the amino acids 139 to 150 and 235 to 239, respectively. N_146_E seemed to be the amino acid most often affected and alternative alterations and combinations thereof seemed to result in conformational changes of the non-penicillin-binding domain eventuating in increased MICs [36,37]. This conclusion is in agreement with results from previous studies focusing on ceftaroline and ceftobiprole resistance, where the amino acid alterations N_146_K and E_150_K, as well as E_150_K and E_239_K, or E_237_K have been associated with an increase in resistance towards the different ß-lactams [17,36–38].

### Conclusions

In contrast to clinically relevant MSSA and MRSA lineages from Germany clonal lineages ST228 and ST239 from the same geographic region were shown to be associated with significantly higher CPT MICs indicating a high proportion of resistant isolates in these lineages. The number of isolates from highly prevalent MRSA lineages in Germany with CPT MICs close to the current EUCAST breakpoint is comparably high and hampers CPT resistance diagnostics as well as surveillance of CPT resistance development. Further studies are essential to (i) establish geographically representative strain collections as a basis for the determination of a “real” CPT MIC wildtype distribution; (ii) to determine the clinical relevance of “borderline” resistant isolates, especially in highly prevalent clonal lineages and (iii) to re-evaluate current guidelines in consideration of additional susceptibility, clinical and pharmacological data in order to confirm the clinical breakpoint set.

## Supporting Information

S1 Table Characterisation of isolates investigated in this study. (XLSX)

S2 Table Primers for detection and characterization of the *mec* genes. (XLSX)

S3 Table Amino acid alterations found in *mecA* of ST228 and ST239 isolates. (XLSX)

## References

[1] Strommenger B, Bartels MD, Kurt K, Layer F, Rohde SM, Boye K et al Evolution of methicillin-resistant *Staphylococcus aureus* towards increasing resistance. J Antimicrob Chemother. 2014; 69: 616–622. 10.1093/jac/dkt413 24150844

[2] Köck R, Schaumburg F, Mellmann A, Koksal M, Jurke A, Becker K et al Livestock-associated methicillin-resistant *Staphylococcus aureus* (MRSA) as causes of human infection and colonization in Germany. PLoS One. 2013; 8: e55040 10.1371/journal.pone.0055040 23418434 PMC3572123

[3] Bush K, Courvalin P, Dantas G, Davies J, Eisenstein B, Huovinen P et al Tackling antibiotic resistance. Nat Rev Microbiol. 2011; 9: 894–896. 10.1038/nrmicro2693 22048738 PMC4206945

[4] Zhanel GG, Sniezek G, Schweizer F, Zelenitsky S, Lagace-Wiens PR, Rubinstein E et al Ceftaroline: a novel broad-spectrum cephalosporin with activity against meticillin-resistant *Staphylococcus aureus* . Drugs. 2009; 69: 809–831. 10.2165/00003495-200969070-00003 19441869

[5] Bush K. Improving known classes of antibiotics: an optimistic approach for the future. Curr Opin Pharmacol. 2012; 12: 527–534. 10.1016/j.coph.2012.06.003 22748801

[6] Moisan H, Pruneau M, Malouin F. Binding of ceftaroline to penicillin-binding proteins of *Staphylococcus aureus* and *Streptococcus pneumoniae* . J Antimicrob Chemother. 2010; 65: 713–716. 10.1093/jac/dkp503 20097788

[7] Clinical and Laboratory Standards Institute (CLSI). Performance standards for antimicrobial susceptibility testing; Twenty-third informational supplement. 2013; M100–S23. Wayne, PA, USA

[8] European Committee on Antimicrobial Susceptibility Testing (EUCAST). Clinical breakpoints—bacteria v 3.1. 2013. Available: http://www.eucast.org/antimicrobial_susceptibility_testing/previous_versions_of_tables/. Accessed 2015 April 9.

[9] European Committee on Antimicrobial Susceptibility Testing (EUCAST). Clinical breakpoints—bacteria v 4.0. 2014. Available: http://www.eucast.org/clinical_breakpoints/. Accessed 2015 April 9.

[10] Sader HS, Flamm RK, Farrell DJ, Jones RN. Activity analyses of staphylococcal isolates from pediatric, adult, and elderly patients: AWARE Ceftaroline Surveillance Program. Clin Infect Dis. 2012; 55 Suppl 3: S181–186. 10.1093/cid/cis560 22903950

[11] Farrell DJ, Flamm RK, Sader HS, Jones RN. Spectrum and potency of ceftaroline tested against leading pathogens causing skin and soft-tissue infections in Europe (2010). Int J Antimicrob Agents. 2013; 41: 337–342. 10.1016/j.ijantimicag.2012.12.013 23466338

[12] Sader HS, Flamm RK, Jones RN. Antimicrobial activity of ceftaroline and comparator agents tested against bacterial isolates causing skin and soft tissue infections and community-acquired respiratory tract infections isolated from the Asia-Pacific region and South Africa (2010). Diagn Microbiol Infect Dis. 2013; 76: 61–68. 10.1016/j.diagmicrobio.2013.01.005 23535208

[13] Zhang H, Xiao M, Yang QW, Wang Y, Wang H, Zhao Y et al High ceftaroline non-susceptibility in *Staphylococcus aureus* isolated from acute skin infections in 15 tertiary hospitals in China. J Med Microbiol. 2013; 62: 496–497. 10.1099/jmm.0.052522-0 23180478

[14] Layer F, Cuny C, Strommenger B, Werner G, Witte W. Current data and trends on methicillin-resistant *Staphylococcus aureus* (MRSA). Bundesgesundheitsblatt Gesundheitsforschung Gesundheitsschutz. 2012; 55: 1377–1386. 10.1007/s00103-012-1560-x 23114436

[15] Schaumburg F, Kock R, Mellmann A, Richter L, Hasenberg F, Kriegeskorte A et al Population dynamics among methicillin-resistant *Staphylococcus aureus* isolates in Germany during a 6-year period. J Clin Microbiol. 2012; 50: 3186–3192. 10.1128/JCM.01174-12 22814464 PMC3457438

[16] Garcia-Alvarez L, Holden MT, Lindsay H, Webb CR, Brown DF, Curran MD et al Meticillin-resistant *Staphylococcus aureus* with a novel *mec*A homologue in human and bovine populations in the UK and Denmark: a descriptive study. Lancet Infect Dis. 2011; 11: 595–603. 10.1016/S1473-3099(11)70126-8 21641281 PMC3829197

[17] Mendes RE, Tsakris A, Sader HS, Jones RN, Biek D, McGhee P et al Characterization of methicillin-resistant *Staphylococcus aureus* displaying increased MICs of ceftaroline. J Antimicrob Chemother. 2012; 67: 1321–1324. 10.1093/jac/dks069 22398650

[18] Alm R, Lahiri S, Sader HS, Iaconi J. Molecular characterisation of *Staphylococcus aureus* isolates that are non-susceptible to ceftaroline isolated during the 2010 surveillance programme ECCMID—European Congress of Clinical Microbiology and Infectious Diseases 2013 Berlin.

[19] Strommenger B, Braulke C, Heuck D, Schmidt C, Pasemann B, Nübel U et al *spa* Typing of *Staphylococcus aureus* as a frontline tool in epidemiological typing. J Clin Microbiol. 2008; 46: 574–581. 18032612 10.1128/JCM.01599-07 PMC2238071

[20] Enright MC, Day NP, Davies CE, Peacock SJ, Spratt BG. Multilocus sequence typing for characterization of methicillin-resistant and methicillin-susceptible clones of *Staphylococcus aureus* . J Clin Microbiol. 2000; 38: 1008–1015. 10698988 10.1128/jcm.38.3.1008-1015.2000 PMC86325

[21] Uhlemann AC, Otto M, Lowy FD, DeLeo FR. Evolution of community- and healthcare-associated methicillin-resistant *Staphylococcus aureus* . Infect Genet Evol. 2014; 21: 563–574. 10.1016/j.meegid.2013.04.030 23648426 PMC3884050

[22] Nimmo GR, Bergh H, Nakos J, Whiley D, Marquess J, Huygens F et al Replacement of healthcare-associated MRSA by community-associated MRSA in Queensland: confirmation by genotyping. J Infect. 2013; 67: 439–447. 10.1016/j.jinf.2013.07.020 23872210

[23] MacGowan AP, Noel AR, Tomaselli S, Bowker KE. Pharmacodynamics of ceftaroline against *Staphylococcus aureus* studied in an in vitro pharmacokinetic model of infection. Antimicrob Agents Chemother. 2013; 57: 2451–2456. 10.1128/AAC.01386-12 23459495 PMC3716170

[24] Richter SS, Heilmann KP, Dohrn CL, Riahi F, Costello AJ, Kroeger JS et al Activity of ceftaroline and epidemiologic trends in *Staphylococcus aureus* isolates collected from 43 medical centers in the United States in 2009. Antimicrob Agents Chemother. 2011; 55: 4154–4160. 10.1128/AAC.00315-11 21709080 PMC3165333

[25] Kim C, Milheirico C, Gardete S, Holmes MA, Holden MT, de Lencastre H et al Properties of a novel PBP2A protein homolog from *Staphylococcus aureus* strain LGA251 and its contribution to the beta-lactam-resistant phenotype. J Biol Chem. 2012; 287: 36854–36863. 10.1074/jbc.M112.395962 22977239 PMC3481288

[26] Canton Moreno R, Livermore DM, Morosini MI, Díaz-Regañón J, Pascual E, Ampudia et al on behalf of the PREMIUM study group. Etest versus broth microdilution for ceftaroline minimum inhibitory concentration (MIC) determination for Gram-positive organisms: results from PREMIUM, a European study ECCMID—European Congress of Clinical Microbiology and Infectious Diseases 2013 Berlin.

[27] Campanile F, Bongiorno D, Borbone S, Stefani S. Hospital-associated methicillin-resistant *Staphylococcus aureus* (HA-MRSA) in Italy. Ann Clin Microbiol Antimicrob. 2009; 8: 22 10.1186/1476-0711-8-22 19552801 PMC2708121

[28] Mick V, Dominguez MA, Tubau F, Linares J, Pujol M, Martin R. Molecular characterization of resistance to Rifampicin in an emerging hospital-associated Methicillin-resistant *Staphylococcus aureus* clone ST228, Spain. BMC Microbiol. 2010; 10: 68 10.1186/1471-2180-10-68 20202188 PMC2844403

[29] Conceicao T, Aires-de-Sousa M, Fuzi M, Toth A, Paszti J, Ungvari E et al Replacement of methicillin-resistant *Staphylococcus aureus* clones in Hungary over time: a 10-year surveillance study. Clin Microbiol Infect. 2007; 13: 971–979. 17697003 10.1111/j.1469-0691.2007.01794.x

[30] Krziwanek K, Luger C, Sammer B, Stumvoll S, Stammler M, Sagel U et al MRSA in Austria—an overview. Clin Microbiol Infect. 2008; 14: 250–259. 18070133 10.1111/j.1469-0691.2007.01896.x

[31] Vogel V, Falquet L, Calderon-Copete SP, Basset P, Blanc DS. Short term evolution of a highly transmissible methicillin-resistant *Staphylococcus aureus clone* (ST228) in a tertiary care hospital. PLoS One. 2012; 7: e38969 10.1371/journal.pone.0038969 22720005 PMC3377700

[32] Leopold SR, Goering RV, Witten A, Harmsen D, Mellmann A. Bacterial whole-genome sequencing revisited: portable, scalable, and standardized analysis for typing and detection of virulence and antibiotic resistance genes. J Clin Microbiol. 2014; 52: 2365–2370. 10.1128/JCM.00262-14 24759713 PMC4097726

[33] Alp E, Klaassen CH, Doganay M, Altoparlak U, Aydin K, Engin A et al MRSA genotypes in Turkey: persistence over 10 years of a single clone of ST239. J Infect. 2009; 58: 433–438. 10.1016/j.jinf.2009.04.006 19446883

[34] Xiao M, Wang H, Zhao Y, Mao LL, Brown M, Yu YS et al National surveillance of methicillin-resistant *Staphylococcus aureus* in China highlights a still-evolving epidemiology with 15 novel emerging multilocus sequence types. J Clin Microbiol. 2013; 51: 3638–3644. 10.1128/JCM.01375-13 23985906 PMC3889745

[35] Nickerson EK, West TE, Day NP, Peacock SJ. *Staphylococcus aureus* disease and drug resistance in resource-limited countries in south and east Asia. Lancet Infect Dis. 2009; 9: 130–135. 10.1016/S1473-3099(09)70022-2 19179228

[36] Banerjee R, Gretes M, Basuino L, Strynadka N, Chambers HF. In vitro selection and characterization of ceftobiprole-resistant methicillin-resistant *Staphylococcus aureus* . Antimicrob Agents Chemother. 2008; 52: 2089–2096. 10.1128/AAC.01403-07 18378703 PMC2415812

[37] Alm RA, McLaughlin RE, Kos VN, Sader HS, Iaconis JP, Lahiri SD. Analysis of *Staphylococcus aureus* clinical isolates with reduced susceptibility to ceftaroline: an epidemiological and structural perspective. J Antimicrob Chemother. 2014; 69: 2065–2075. 10.1093/jac/dku114 24777906

[38] Katayama Y, Zhang HZ, Chambers HF. PBP 2a mutations producing very-high-level resistance to beta-lactams. Antimicrob Agents Chemother. 2004; 48: 453–459. 14742194 10.1128/AAC.48.2.453-459.2004 PMC321522

